# Metabolites and MRI-Derived Markers of AD/ADRD Risk in a Puerto Rican Cohort

**DOI:** 10.21203/rs.3.rs-3941791/v1

**Published:** 2024-02-13

**Authors:** Scott Gordon, Jong Soo Lee, Tammy M. Scott, Shilpa Bhupathiraju, Jose Ordovas, Rachel S. Kelly, Rafeeque Bhadelia, Bang-Bon Koo, Sherman Bigornia, Katherine L. Tucker, Natalia Palacios

**Affiliations:** University of Massachusetts Lowell; University of Massachusetts Lowell; Tufts University; Harvard School of Public Health; Tufts University; Harvard Medical School; Harvard Medical School; University of Massachusetts Lowell; University of Massachusetts Lowell

**Keywords:** Metabolomics, Cognitive Decline, Latinos, Puerto Rican

## Abstract

**Objective:**

Several studies have examined metabolomic profiles in relation to Alzheimer’s disease and related dementia (AD/ADRD) risk; however, few studies have focused on minorities, such as Latinos, or examined Magnetic-Resonance Imaging (MRI)-based outcomes.

**Methods:**

We used multiple linear regression, adjusted for covariates, to examine the association between metabolite concentration and MRI-derived brain age deviation. Metabolites were measured at baseline with untargeted metabolomic profiling (Metabolon, Inc). Brain age deviation (BAD) was calculated at wave 4 (~ 9 years from Boston Puerto Rican Health Study (BPRHS) baseline) as chronologic age, minus MRI-estimated brain age, representing the rate of biological brain aging relative to chronologic age. We also examined if metabolites associated with BAD were similarly associated with hippocampal volume and global cognitive function at wave 4 in the BPRHS.

**Results:**

Several metabolites, including isobutyrylcarnitine, propionylcarnitine, phenylacetylglutamine, phenylacetylcarnitine (acetylated peptides), p-cresol-glucuronide, phenylacetylglutamate, and trimethylamine N-oxide (TMAO) were inversely associated with brain age deviation. Taurocholate sulfate, a bile salt, was marginally associated with better brain aging. Most metabolites with negative associations with brain age deviation scores also were inversely associations with hippocampal volumes and wave 4 cognitive function.

**Conclusion:**

The metabolites identifiedin this study are generally consistent with prior literature and highlight the role of BCAA, TMAO and microbially derived metabolites in cognitive decline.

## INTRODUCTION

It is estimated by the World Health Organization that 55 million people have dementia worldwide, and this number is projected to rise to 75 million by 2030 and 139 million by 2050, as the proportion of the older population (over 65 y) increases (1). Racial disparities impact the risk of developing dementia (2). Compared to non-Hispanic White individuals, the Hispanic population in the United States is disproportionately more affected by dementia, and, among Latinos, Puerto Rican are at higher risk compared to Mexican Americans.(3, 4). While many genetic factors of dementia have been identified the influence of metabolomic factors has yet to be fully determined.

Several research studies have been conducted so far to investigate the connection between blood metabolites and the risk of Alzheimer’s disease (AD) (5–8). The identification of biomarkers associated with cognitive decline is crucial for early diagnosis and potential treatment of AD/ADRD. Recent studies have reported that metabolic pathways responsible for lipid processing, immune function, phagocytosis, and neurotransmitter function, are altered in patients with dementia (4, 8), and associations blood metabolites and cognitive decline (5, 8, 9). A recent study of blood and post-mortem brain tissue samples in longitudinal cohorts of aging and dementia identified that elevated levels of acylcarnitines predicted a significantly lower risk of AD. In contrast, the presence of serum metabolites, including such as glycerophospholipids and asparagine, was associated with a decline in global cognition (8).

Recent research has shown that there may be a relationship brain structural changes and metabolite alterations in AD/ADRD (7, 10–12). For example, lower serum levels of 7α-hydroxycholesterol (7α-OHC) and primary BAs were found to be associated with higher brain amyloid deposition, faster white matter lesions accumulation, and faster brain atrophy (7). Another study found that individuals with MCI and AD had lower levels of triglycerides with long chain polyunsaturated fatty acids compared to cognitively normal individuals, and the scores of these triglycerides were also associated with hippocampal volume and entorhinal cortical thickness (13). In participants with the APOE ε4 allele, these triglycerides were further associated with cerebrospinal fluid β-amyloid1–42 values and entorhinal cortical thickness (13).There have been few studies on cognitive decline and metabolomics that included diverse participants, especially Latinos. Further, none such studies used an untargeted approach, which could reveal novel associations and the findings have been inconsistent (14, 15). Puerto Ricans have metabolic and lifestyle risk factors that predispose them to dementia risk, such as high prevalence of diabetes, hypertension, and poorer diet quality. We have shown that diabetes and hypertension are associated with brain imaging measures related to AD/ADRD (16). In a recent analysis conducted within the Study of Latinos-Investigation of Neurocognitive Aging (SOL-INCA), several metabolites, such as phosphatidylcholines, were associated with cognitive decline (17). Previous cross-sectional analysis in Boston Puerto Rican Health Study (BPRHS) cohort reported that plasma concentration of β-cryptoxanthin was positively, and of N-acetylisoleucine and tyramine O-sulfate negatively, associated with cognition (18). In the present study, we report the findings of brain imaging studies and blood metabolites collected as part of the BPRHS cohort.

## METHODS

The Boston Puerto Rican Health Study (BPRHS) is a longitudinal study of 1500 Puerto Rican adults residing in the greater Boston area who were recruited starting in 2004 and followed prospectively. Four study visits have been conducted to date. Metabolomic profiling was conducted at study baseline. Participants involved in this study were initially identified from the year 2000 census. Participants were chosen from the greater Boston area in neighborhoods densely populated by Hispanic individuals (~ 80%) and were contacted via large Puerto Rican events within the area (9%) or through personal referrals or media. The participants were between 45 and 75 years at baseline. Some participants were excluded due to severe health conditions, reports of plans to move away from the area, or a Mini-Mental State Examination (MMSE) score of less than or equal to 10 at baseline. The National Health and Nutrition Examination Survey (NHANES III), the Hispanic Health and Nutrition Examination Survey (HHANES), and the National Health Interview Survey Supplement on Aging (NHIS) were used to design a questionnaire to determine the demographic and socio-economic status of participants. Questions were asked on level of education as well as household income. A food frequency questionnaire (FFQ) was designed for this population and was used to assess the participants’ dietary intake (19). Physical activity was assessed using a modified Paffenbarger questionnaire. Blood samples were drawn after a 12 h fast and immediately transported on ice to the Human Nutrition Research Center on Aging at Tufts University. Blood samples were kept at 4°C and separated within 2 h in a refrigerated centrifuge. The separated plasma samples were aliquoted into 1 mL cryogenic screw-cap tubes and stored at − 80°C.

Metabolomic profiling was conducted at baseline. Approximately 12.7 years later at visit 4, brain MRI was conducted. All participants who consented during the 4th BPRHS wave were eligible for MRI. 169 participants had both baseline metabolomic and MRI data and formed the analytic sample for this study.

Assessment of MRI-based outcomes: In this analysis, we focused on four outcomes related to AD/ADRD risk: brain volume, hippocampal volume, and global cognitive function (GCS). We considered brain age deviation as our primary outcome. Briefly, T1-weighted structural imaging data were obtained with a GE 3T MRI scanner for each participant’s cortical thickness, area, volume, and cerebellar-subcortical and cortical summary statistics. Detailed automated hippocampal segmentation was included in our processing pipeline using FreeSurfer v6.0.25. We further extracted the volumes of the left and right hippocampus and total intracranial volume (ICV). Brain age was calculated via a machine learning model(20), whether the raw brain age deviation score (chronologic age – brain age) represents the rate of biological brain aging relative to chronologic age. The raw deviation values were then converted to z scores. Hippocampal volume and global cognitive function were used as supporting outcomes. To account for interindividual variation in brain morphology and possible measurement errors that may be introduced by movement artifact and machine-dependent MR image intensity scales, the hippocampal volumes were normalized by dividing the raw hippocampal volume by the total intracranial volume (ICV) as described in (20, 21). The brain age deviation score was estimated as described in detail in

Assessment of supporting outcome: global cognitive function score: To examine whether metabolites associated with MRI-based outcomes were also associated cognition, we used a supplemental outcome, global cognitive function composite score (GSC). It was calculated (22) based on a series of neuropsychological tests that were conducted in the participant’s language of choice (98% in Spanish) and included (1) a Mini-Mental State Examination (MMSE, general cognition) (23); a 16-word list learning task for verbal memory with (2) word list learning (sum of words recalled over 5 attempts), (3) word recognition and (4) percentage retention (# of words recalled after a delay relative to # of correct responses on the fifth learning trial); (5) digit span forward and backward (working memory) (24); (6) Stroop test (executive function) for processing speed (24), cognitive flexibility, and response to inhibition, and (7) verbal fluency (naming as many words as possible starting with a given letter) (24); (8) clock drawing (25); and (9) figure copying (possible range: 0–27) to measure visual-spatial organization (26). Individual test scores were converted to z scores, and a global cognitive function composite score (GCS) was calculated as the arithmetic mean of the individual z scores. The GCS was used as the outcome variable in this analysis. If a participant did not complete an individual test, the given score was imputed using the minimum z score of the same individual test for the rest of the cohort. If the missing values were due to illiteracy, hearing impairment, or poor vision, the available recorded individual test values were averaged. The GSC for visit 4 was used in the analyses.

Assessment of Metabolites: Metabolon (Metabolon, Inc., Morrisville, NC) performed the Metabolomic profiling for the participants, using previously described proprietary procedures (27). At baseline, participants provided fasting blood samples, which were processed as described above and subsequently stored at −80C until profiling. Briefly, the samples were analyzed using liquid chromatography-MS/MS with positive and negative ion modes (Waters ACQUITY ultra-performance liquid chromatography; Thermo Scientific Q-Exactive high resolution/accurate mass spectrometer interfaced with a heated electrospray ionization source and Orbitrap mass analyzer operated at 35,000 mass resolution). Metabolites were separated and quantified via four modes of sampling: 1) acidic positive ion (hydrophilic molecules); 2) acidic positive ion (hydrophobic molecules); 3) basic negative ion; and 4) negative ionization from eluent of a HILIC column. More than 3,300 commercially available purified molecules were used as reference for peak identification. The injection order was random and internal quality control samples were included. Relative metabolite concentration was reported as a normalized area under the curve. From this data provided by Metabolon, 1,303 metabolites were identified, 943 of which were assigned a chemical annotation, and the other 360 were unknown. We restricted this analysis to the 943 known metabolites in order to focus on known biological function, and excluded xenobiotics. We further excluded metabolites that were available for fewer than 20% of the participants. Missing values were imputed for individual metabolites as half of the minimum value of the respective metabolite across participants with non-missing values (18). Metabolites were log-transformed and Pareto-scaled (28). After imputation and processing the data, this analysis included 621 metabolites.

Models were adjusted for age, sex, hypertension (yes/no), education, BMI, smoking status, physical activity score, diabetes (yes/no), APOE genotype, Mediterranean diet score, use of proton pump inhibitor (PPI), metformin and non-steroidal inflammatory medications, as assessed at baseline. Age was modeled as a continuous covariate and education \as a categorical covariate (< 8th grade, 9th −12th grade, college/graduate school). Frequency and history of smoking were assessed as current, former, or never. Body mass index (BMI) was included as a continuous covariate, determined as weight (kg) divided by height (m) squared. Participants were considered to have diabetes if they had a fasting glucose > = 126 mg/dL or reported using diabetes medication. Hypertension was defined as systolic blood pressure of 140 or greater, diastolic blood pressure of 90 mm Hg, or hypertension medication use. The APOE4 covariate, defined as the presence of at least one copy of the e4 allele), was assessed via Genome-Wide Association Testing (GWAS). Mediterranean diet adherence score was assessed using Food Frequency Questionnaire (FFQ) data and treated as a continuous covariate in the analyses, with a range of 1 (low adherence) to 9 (high adherence) (29).

### Statistical Analysis:

We used multiple linear regression to examine the association between metabolite concentration at baseline and brain age deviation. Analogous analyses were conducted for supporting outcomes: normalized hippocampal volume, as well as global cognitive score at wave 4 (year 12 follow-up). For metabolites associated with brain age deviation, g we also examined their association with secondary outcomes: hippocampal volume and IGCS. All analyses were performed using R statistical software (version 4.2.1).

We computed Spearman correlation coefficients between the top metabolites associated with BAD, to visualize the inter-relationships. All *P* values were corrected for multiple comparisons using the Benjamini-Hochberg False Discovery Rate (FDR).

## RESULTS

Most of the study participants were women (80.5%), with mean ± SD age of 54.8 ± 6.2 years and average BMI of 32.8 ± 6.9 kg/m2 (Table 1). More than half (52.5%) were current smokers, 30.7% had type 2 diabetes. And 40.2% had an education equivalent to 8th grade or less.

**Figure 2:**
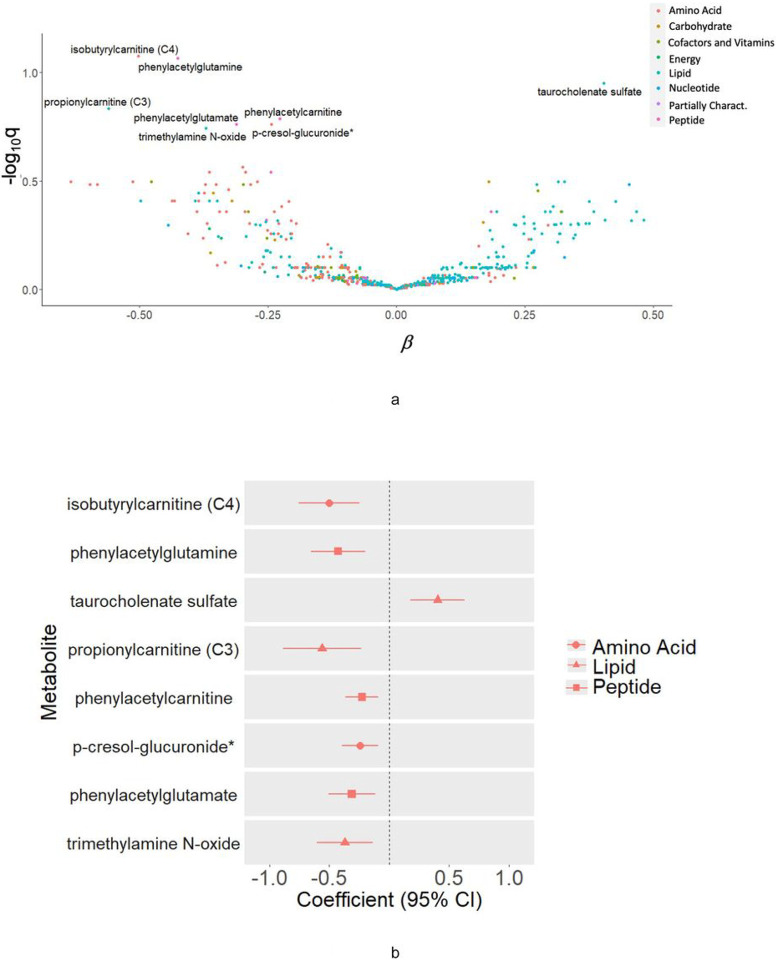
A. Volcano plot of metabolites associated with brain age deviation among 169 BPRHS participants with MRI and untargeted metabolomic measures. B. Top metabolites, associated with brain age deviation in BPRHS (nominal p<0.05), by metabolite class.

Higher levels of several carnitines, including isobutyrylcarnitine (C4) (ß = −0.449, 95%CI = [−0.675,−0.223], P = 0.0026, pFDR = 0.084), propionylcarnitine (C3), (ß = −0.500, 95%CI = [−0.793,−0.208], P = 0.018, pFDR = 0.15), and phenylacetylcarnitine (ß = −0.203, 95%CI = [−0.325,−0.081], P = 0.025, pFDR = 0.16), ß = −0.380, 95%CI = [−0.582,−0.179], P = 0.0053, pFDR = 0.086) were associated with detrimental brain aging in BPHRS. Higher levels of trimethylamine N-oxide (TMAO) (ß = −0.330, 95%CI = [−0.541,−0.120], *P* = 0.044, pFDR = 0.18) were also associated with worse brain aging. Likewise, phenylacetylglutamine (ß = −0.380, 95%CI = [−0.582,−0.179], P = 0.0053, pFDR = 0.086), phenylacetylglutamate (ß =−0.278, 95%CI = [−0.452,−0.104], *P* = 0.17, pFDR = 0.037) and p-cresol-glucuronide (ß = −0.217, 95%CI = [−0.352,−0.082], *P* = 0.034, pFDR = 0.17) were associated with worse brain aging (Fig. 2 and Supplemental Table 1). One metabolite, a bile acid taurine conjugate, taurocholenate sulfate (ß = 0.403, 95%CI = [0.178,0.628], P = 0.010, pFDR = 0.11) was marginally positively associated with brain age deviation (Fig. 2 and Supplemental Table 1). No metabolites were significantly associated with hippocampal volume and none were positively associated with cognitive function (Supplemental Table 2).

In secondary analyses focusing on global cognitive function as the outcome, a group of steroid lipid metabolites, including glycolithocholate sulfate (ß = −0.231, 95%CI = [−0.118, 0.020], P = 0.020, pFDR = 0.0018), deoxycholate (ß = −0.205, 95%CI = [−0.313,−0.097], *P* = 0.031, pFDR = 0.0050), taurolithocholate 3-sulfate (ß = 0.206, 95%CI = [0.317,−0.096], *P* = 0.032, pFDR = 0.0058), taurodeoxycholate (ß = −0.174, 95%CI = [−0.273,−0.075], *P* = 0.059, pFDR = 0.013), glycodeoxycholate (ß =−0.151, 95%CI = [−0.239,−0.062], *P* = 0.074, pFDR = 0.018), and p-cresol-glucuronide (ß =−0.218, 95%CI = [−0.330,−0.106], *P* = 0.030, pFDR = 0.0034) were negatively associated with cognitive function at wave 4 (Supplemental Table 2). Higher levels of a diacylglycerol lipid, linoleoyl-arachidonoyl-glycerol (18:2/20:4) (ß = −0.226, 95% CI = (−0.368,−0.084), *P* = 0.038, pFDR = 0.14) were also associated with worse cognitive function at BPHRS wave 4. Figure 2 shows the overall metabolite associations in relation to brain age deviation as volcano plot (2A), as well as estimates for the top metabolites (2B) associated with cognition (nominal p < 0.05).

**Figure 3:**
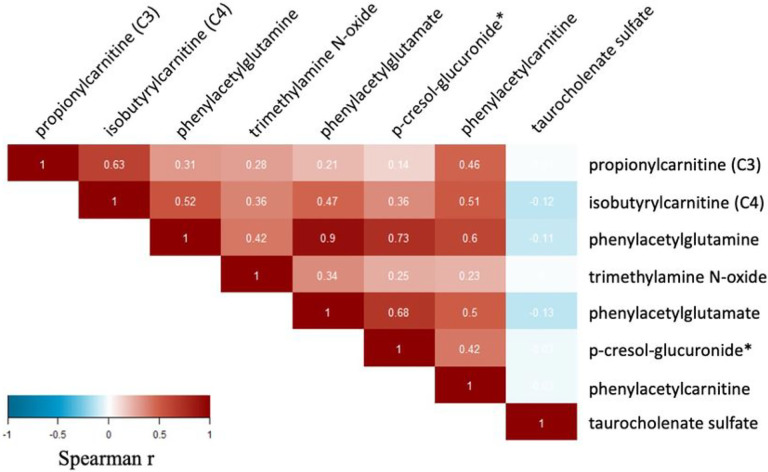
Correlations between metabolites significantly (nominal p<0.05) associated with brain age deviation in BPRHS.

In a correlation analysis between the metabolites associated with brain age and cognitive function (Fig. 3), the strongest intercorrelation was observed between phenylacetylglutamate and phenylacetylglutamine (0.9), both linked to the metabolism of acetylated peptides, as well as a by-product of tyrosine metabolism p-cresol glucuronide with phenylacetylglutamate (r = 0.68) and phenylacetylglutamine (r = 0.73). Correlations between the brain-age associated metabolite are shown in Fig. 3.

**Figure 4:**
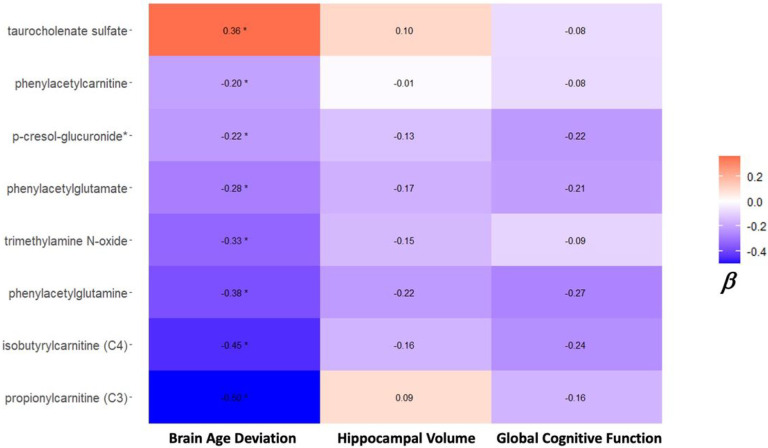
Top metabolites associated with brain age in BPHRS, have similar, although less strong and not significant, associations with hippocampal volume and global cognitive function. Dispalyed are beta coefficients for the association with each outcome. *designates significant at nominal p<0.05 level.

Most metabolites negatively associated with brain age deviation score, were similarly, although not significantly, associated with cognitive function and hippocampal volumes (Fig. 4). For example, the short-chain carnitine derivatives, isobutyrylcarnitine (C4) propionylcarnitine (C3), phenylacetylglutamine and phenylacetylglutamate, were negatively associated with cognition a hippocampal volume at wave 4. Conversely, taurocholenate sulfate was positively associated with brain age and hippocampal volume, but not with cognition and brain volume outcomes.

## DISCUSSION

In this study, we examined the relationship between metabolites and brain imaging markers of AD/ADRD, and identified several metabolites, including secondary BA, BCAA and TMAO associated with these AD/ADRD markers in older Boston Area Puerto Rican adults.

Our results indicate a potential inverse association between trimethylamine-N-oxide (TMAO) on brain aging and hippocampal volume. TMAO is biosynthesized from choline, betaine, and L-carnitine by gut microbial metabolism. Blood TMAO increases after consumption of foods such as fish, eggs and red meat that are rich in L-carnitine and phosphatidylcholine, and recent studies have linked circulating TMAO to inflammation, type 2 diabetes, and cardiovascular disease (30, 31). The influence of TMAO on dementia risk is still under debate; however, TMAO can cross the blood-brain barrier, and high TMAO concentration can induce neuroinflammation by triggering proinflammatory signaling pathways via NF-kB and cytokines (31). Past studies have reported that AD and MCI patients had elevated TMAO in their CSF, suggesting that this metabolite plays a role in oxidative stress and may contribute to neurodegeneration (32). Our findings further corroborate the connection and potential negative role of TMAO in aging, inflammation, and AD/ADRD risk.

We identified inverse associations between two short-chain acyl carnitines, propionyl carnitine (C3) and isobutylryl carnitine (C4 and brain age, as well as phenylacetyl carnitine, intermediate metabolites of amino acid and fatty acid oxidation in mitochondria which have received attention for their potential use as biomarkers for different disorders, including schizophrenia (33, 34). Disruptions in metabolic profiles of acylcarnitines have been observed in Alzheimer’s disease and MCI(6). Propionyl carnitine (C3) is biosynthesized from propionyl-Co, a metabolite of branched-chain aminos acids (isoleucine, valine, threonine, methionine), odd-chain fatty acids, and side chains of cholesterol. Propionylcarnitine has been previously observed to be elevated in the blood of participants with obesity, type 2 diabetes (35), and heart failure (36). Similarly, isobutyryl carnitine (C4) is a product of the metabolic oxidation of valine and fatty acids. The accumulation of C3-, C4- and C5-acylcarnitines could be due to incomplete oxidation of fatty acids and branched-chain amino acids at the level of different acyl-CoA dehydrogenase enzyme (37). There is mounting evidence on the role of branched-chain amino acids in brain function and AD/ADRD risk (38). A recent study of metabolomic profiles in the Alzheimer’s Disease Neuroimaging Initiative cohort reported an association of propionylcarnitine with decreased amyloid-β accumulation and higher memory scores (6).

Our results support the previously observed link between bile acid metabolites and AD/ADRD. Table 2 shows elevated blood levels of six cholesterol derivatives (glycine and taurine conjugated forms) involved in secondary bile acid metabolism, and of glycochenodeoxycholate glucuronide, involved in primary bile acid metabolism, each of which were associated with negative cognitive trajectory. Bile acids are produced in the liver from cholesterol and are further processed by gut bacteria (4). Bile acids can cross the blood-brain barrier, bind to nuclear receptors, and initiate important signaling and regulatory responses. A dysregulated bile acid profile is associated with cognitive impairment and Alzheimer’s Disease (AD). A recent study comparing blood metabolites of Alzheimer’s Disease patients to those of cognitively normal older adults reported significantly lower serum concentration of primary bile acids (cholic acid CA) and increased concentrations of the bacterially produced, secondary BA, deoxycholic acid (DCA), and its glycine and taurine conjugated forms (4). In addition, several mutations in the genes involved in immune response were observed in AD profiles and showed associations with cholesterol metabolism (39) and proper neuronal myelination (40). A secondary bile salt, sodium taurocholate, showed a positive association with cognition in our study. Sodium taurocholate sulfate is a bile salt that forms micelles around insoluble lipids, such as cholesterol, for their transport. The sodium/taurocholate cotransporting peptide (NTCP) is one of the key basolateral transporter proteins that maintain bile salt homeostasis and is highly expressed in brain tissue (41).

Another metabolite identified in this study is p-cresol glucuronide, a gut microbial co-metabolite of amino acids phenylalanine and tyrosine, formed via p-cresol. While one recent study found that it promotes blood-brain barrier integrity in mice (42), other studies report this metabolite as a uremic toxin linked to cardiac-related mortality and chronic kidney disease (43). Moreover, a recent study of metabolic profiles of bladder cancer suggested p-cresol-glucuronide as a potential biomarker for bladder cancer detection (44). Our results show that p-cresol glucuronide is correlated to metabolites carrying the phenylacyl group, specifically phenylacetyl glutamate, phenylacetyl carnitine and phenylacetyl glutamine. The most studied of this group is a microbial derived metabolite phenylacetyl glutamine. Multiple studies suggest that phenylacetyl glutamine plays an important role in brain and other physiological processes, and that it could be a biomarker in acute ischemic stroke (45) and type 2 diabetes (46).

Several metabolites identified in this study have been previously linked with diabetes and metabolic syndrome, including BCAA and TMAO. Prior studies with the BPRHS cohort reported that the participants had higher prevalence of obesity and age-related conditions, such as type 2 diabetes and hypertension, than those in the general population. Growing evidence shows that diabetes predisposes to structural brain abnormalities and cognitive decline, leading to dementia (47). A recent BPRHS study involving brainMRI imaging reported a potential impact of comorbid diabetes and hypertension on accelerated brain aging and cognitive impairment, with overall declines in executive function and global cognitive function (16). Moreover, the smallest hippocampal volumes and larger brain age deviations were observed among the participants with type 2 diabetes and hypertension.

Several metabolites were significantly associated with AD/ADRD outcomes in our study, including secondary bile acids, TMAO, phenylacetylcarnitine, and p-cresol-glucuronide are microbially derived. There is a close relationship between the host plasma metabolome and gut microbiota. As biochemical nutrients such as proteins, lipids, carbohydrates, etc. are digested and processed in the gut, they are catabolized into host-derived and microbial metabolites. Both types of metabolites can cross into the bloodstream and induce biological responses that affect different processes including those of the central nervous system (31). For example, the levels of microbially derived p-cresol glucuronide and phenylacetylglutamine showed strong associations with certain bacterial species in the gut. Metabolomics reflects the effects of genetic make-up, lifestyle, and environmental factors on a person’s overall health and cognition.

Understanding how the timing of blood metabolites is correlated to the risk of developing a disease has gained much attention in recent years (48, 49). In our study, the blood metabolites were measured on average 12.7 years prior to the brain imaging and cognitive outcomes. Blood metabolite makeup can vary depending on one’s lifestyle and changes in diet and medication over time (49), a limitation of this study. In our previous, cross-sectional report (18) that considered metabolite and cognitive data at baseline in the BPRHS, the key metabolites associated with cognitive function, were somewhat different from the current study, and were primarily sugars. However, MRI was only collected at the wave 4 visit in the BPRHS, so we were not able to examine MRI outcomes cross sectionally. While there are several reports on the long-term biomarker-outcome associations, data on the long-term within-person stability of metabolomics are generally lacking (49, 50). However, between-person metabolite variability tends to be higher than within-person variability. A recent report suggests that within-person metabolite stability over 10 years is reasonable for many lipid and polar metabolites, but can vary by metabolite class (49). The most stable metabolites were nucleotides and analogs, diglyceride, plasmalogens, bile acid precursors, and cholesterol esters, whereas lysophospholipids and some cholesterolderivatives showed lower stability. Diet-related metabolites are likely to have lower within-person stability, e.g. triglycerides with unsaturated fatty acids tend to be more stable over time than triglycerides with saturated fatty acids. In summary, this study of the metabolome and MRI markers of brain aging over 12 y of follow-up in the BPRHS, the first such study in Puerto Ricans, identified several metabolites, including isobutyrylcarnitine, propionylcarnitine, phenylacetylglutamine, phenylacetylcarnitine, p-cresol-glucuronide, and trimethylamine N-oxide (TMAO) as associated facster brain aging, and taurocholate sulfate, a bile salt, was associated with better brain aging. These findings are generally consistent with studies on metabolomics and AD/cognitive decline in other populations. Further research on the role of the metabolome in underserved populations, particularly Latinos, is necessary to understand the mechanisms of the observed associations.

## Figures and Tables

**Figure 1:**
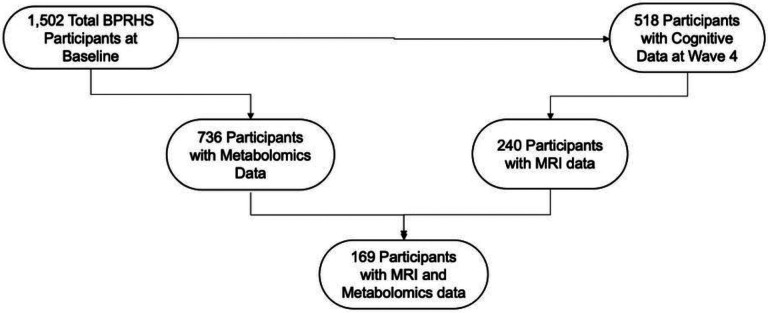
Flowchart for participant inclusion in the study. The study is based on 169 BPRHS participants with MRI and Untargeted Metabolomics Measures.

**Table 1:** Characteristics of BPRHS participants with MRI imaging and metabolomic data included in the study, compared to those without these measures.

	Study subsample (n = 169)	Excluded subsample (n = 1333)
	Mean (SD) / % (n)	Mean (SD) / % (n)
Age (y)	54.8 (6.2)	57.3 (7.71)
Female sex (%)	80.5 (136)	69.2 (923)
Education (< = 8th grade)	40.2 (68)	47.7 (628)
Dietary Quality (Mediterranean Score)	4.37 (1.88)	4.36 (1.60)
Physical Activity Score	32.1 (3.79)	31.5 (4.83)
Body mass index (kg/m^2^)	32.8 (6.94)	31.8 (6.61)
Type 2 diabetes (%)	30.8 (52)	39.9 (532)
Current smoker (%)	52.7 (89)	24.8 (330)
APOE (%e4 allele)	25.6 (41)	18.3 (244)

a *P* value comparing sub-sample with metabolomic profiling to those without profiling

b values presented are mean (sd) for continuous or proportion (N) for categorical variables.

b Center for Epidemiological Studies – Depression scale.

## Data Availability

The data supporting the findings of this study are available on request from the corresponding author. The data are not publicly available due to privacy or ethical restrictions.
